# Nurses’ Perceptions of the Effect of Organizational Support, Role Clarity, and Ambiguity on Career Plateauing: Implications for the Healthcare Workforce

**DOI:** 10.3390/healthcare14060755

**Published:** 2026-03-18

**Authors:** Eman Kamel Hossny, Shimaa Elwardany Aly, Intisar Alsheikh Mohamed, Abeer Mohamed Abdelkader, Aml Sayed Ali Abdelrahem, Fayza M. Mohammed, Taliaa Mohsen Al-Yafeai, Hanan Sayed Younes

**Affiliations:** 1 Community and Mental Health Nursing Department, Faculty of Nursing, Zarqa University, Zarqa 13132, Jordan; 2 Nursing Administration Department, Faculty of Nursing, Assiut University, Assiut 71515, Egypt; hanan.sayed1990@aun.edu.eg; 3 Primary Care Nursing Department, Faculty of Nursing, Al-Ahliyya Amman University, Amman 19328, Jordan; 10653@ammanu.edu.jo; 4 Community Health Nursing, Faculty of Nursing, Assiut University, Assiut 71515, Egypt; 5 Department of Nursing, College of Applied Medical Sciences, University of Jeddah, Jeddah 21589, Saudi Arabia; iaalsheikh@uj.edu.sa; 6 Nursing Education Department, Faculty of Nursing, Minia University, Minia 61519, Egypt; aosman@kfu.edu.sa (A.M.A.); dramlpediatric@yahoo.com (A.S.A.A.); 7 Nursing Department, College of Applied Medical Science, King Faisal University, Al-Ahsa 31892, Saudi Arabia; 8 Community Health Nursing, College of Applied Medical Science, Alula Branch, Taibah University, Alula 43562, Saudi Arabia; fmmohammed@taibahu.edu.sa; 9 Adult Health Nursing, College of Nursing, Ras AlKhaimah Medical and Health Sciences University, Ras Al Khaimah 11172, United Arab Emirates; taliaa@rakmhsu.ac.ae

**Keywords:** career plateauing, organizational support, role clarity, role ambiguity, nurses, university hospitals

## Abstract

**Background:** Career plateauing is an increasingly recognized workforce challenge in nursing, associated with reduced motivation, professional growth, and retention. While organizational support and role-related factors are known to influence nurses’ work outcomes, their combined effect on career plateauing remains insufficiently explored, particularly in university hospital settings. **Aim:** This study aimed to examine the effect of perceived organizational support, role clarity, and role ambiguity on career plateauing among staff nurses working in university hospitals. **Methods:** A descriptive correlational cross-sectional design was employed. Data were collected from 210 staff nurses working in two Egyptian university hospitals using four validated instruments: a demographic data sheet, the Perceived Organizational Support Scale, the Role Clarity and Role Ambiguity Questionnaire, and the Career Plateauing Scale. Data were analyzed using Pearson correlation and univariate and multivariate linear regression analyses with SPSS version 27. **Results:** Most nurses reported moderate levels of organizational support (85.2%) and career plateauing (84.3%). Role ambiguity demonstrated a significant negative correlation with career plateauing (r = −0.441, *p* < 0.01), while organizational support and role clarity were positively associated with plateauing perceptions. Multivariate regression analysis revealed that role ambiguity was the strongest predictor of both hierarchical and job content plateauing (*p* < 0.001), followed by role clarity and organizational support. **Conclusions:** Reducing role ambiguity may alleviate perceptions of career plateauing among nurses. However, increased organizational support and role clarity alone may heighten awareness of limited advancement opportunities if not accompanied by tangible career development pathways. Nursing leaders should focus on clarifying roles while creating realistic and transparent opportunities for professional growth.

## 1. Introduction

Human resources represent one of the most valuable assets within hospital systems, alongside capital, infrastructure, and technology. The effective utilization of human resources plays a crucial role in enhancing healthcare quality and organizational competitiveness. In an increasingly demanding healthcare environment, hospitals strive to maintain an edge by cultivating a well-trained, skilled, and responsive workforce through structured education, training, and development programs [1].

One pivotal factor in this context is organizational support, which significantly shapes employee attitudes and behaviors. When employees perceive that their organization values their contributions and genuinely cares about their well-being, they are more likely to demonstrate positive work behaviors, such as increased commitment, job satisfaction, and proactive engagement. A supportive environment fosters trust and loyalty, encouraging employees to align their goals with those of the organization and exceed role expectations. Furthermore, organizational support acts as a buffer against occupational stress, reduces turnover intentions, and contributes to enhanced performance [2]. It reflects the extent to which employees believe their organization recognizes their efforts and provides them with necessary resources, equitable treatment, and developmental opportunities [3]. In a recent cross-sectional study among Jordanian critical care nurses, perceived organizational support showed a significant positive correlation with job performance, highlighting its essential role in improving care quality and professional outcomes [4].

Closely linked to organizational support are role clarity and role ambiguity, which significantly influence professional functioning, especially in dynamic healthcare settings. Role clarity refers to the degree to which individuals understand their job responsibilities, performance expectations, and how their role integrates within the healthcare team. In nursing practice, this includes clarity regarding one’s duties, colleagues’ roles, and standards of care. High role clarity enhances communication, teamwork, and patient safety, while reducing stress and role conflict [5].

In contrast, role ambiguity arises when nurses lack clear information about their responsibilities or expectations, leading to uncertainty, stress, and reduced effectiveness. Rapid changes in healthcare delivery have expanded and complicated nurses’ roles, often resulting in overlapping tasks and inconsistent or missing information, which exacerbate ambiguity in the workplace [6].

Within this complex professional context, career plateauing has emerged as a significant concern. Nurses often expect progressive career development; however, volatile organizational structures, economic instability, and downsizing have resulted in many remaining in the same positions for extended periods without advancement. Career plateauing occurs when nurses perceive limited opportunities for promotion, while facing heavy workloads and organizational constraints that affect motivation, psychosocial safety climate (PSC), and work engagement (WE) [7,8].

Despite its impact, career plateauing remains underexplored in the nursing literature. Although leadership and organizational factors, such as organizational agility and ambidextrous leadership, can enhance nurses’ readiness for change, career plateauing may undermine motivation and job satisfaction. It typically occurs when individuals remain in the same role for prolonged periods with limited opportunities for career growth. The phenomenon is commonly classified into two main types: hierarchical plateauing, where opportunities for upward mobility are restricted, and job content plateauing, where tasks become repetitive and lack challenge [9,10,11].

Given the strong influence of organizational factors on employee development, it is crucial to investigate how perceived organizational support, role clarity, and role ambiguity collectively contribute to career plateauing among nurses.

Significance of this study:

Like other professionals, nurses may face a stage in their careers where advancement and growth opportunities are limited. When this plateauing persists, it can lead to decreased job satisfaction, disengagement, and decreased professional efficacy. This situation not only affects individual nurses but also has implications for workforce stability, healthcare quality, and the long-term development of the nursing profession.

However, when nurses receive adequate organizational support and have clear role definitions and expectations, they are more likely to feel secure, valued, and motivated in their roles. Addressing issues such as plateauing through supportive organizational strategies can contribute to building a more resilient and effective nursing workforce.

The findings aim to provide healthcare leaders with practical insights to enhance nurse retention, job satisfaction, and professional development in clinical settings.

Aims of this study:

This study aims to investigate the effect of organizational support, role clarity, and role ambiguity on career plateauing among staff nurses working in university hospitals.

Research questions:
  1. What are staff nurses’ perceptions of organizational support, role clarity, role ambiguity, and career plateauing?
  2. What is the relationship between organizational support, role clarity, role ambiguity, and career plateauing among staff nurses?
  3. To what extent do organizational support, role clarity, and role ambiguity predict staff nurses’ career plateauing.

## 2. Materials and Methods

### 2.1. Research Design

A descriptive correlational cross-sectional design was used to explore the relationships among organizational support, role clarity, role ambiguity, and career plateauing among staff nurses.

### 2.2. Setting

The study was conducted at two major university hospitals in Egypt: Heart University Hospital (240 beds) and El Rajhi Liver University Hospital (200 beds). Both hospitals serve as tertiary care and teaching facilities.

In Egypt, multiple educational pathways lead to nursing practice, including secondary nursing schools, technical nursing institutes, and university bachelor’s degree programs. While all pathways allow for entry into clinical nursing roles, they differ in academic preparation, professional expectations, and opportunities for advancement. These differences may influence nurses’ perceptions of career progression and organizational support, as university-educated nurses may hold higher expectations regarding promotion and professional development.

### 2.3. Participants

A convenience sample was recruited from eligible staff nurses working in the selected university hospitals according to predefined inclusion criteria. Inclusion criteria were registered nurses with at least five years of clinical experience and willingness to participate. Nurses holding administrative positions were excluded.

An a priori power analysis using G*Power (version 3.1) indicated that a minimum of 92 participants per hospital was required to detect a medium effect size (f^2^ = 0.15) at a significance level of 0.05 and power of 0.80. The final sample exceeded this requirement.

### 2.4. Data Collection Instruments

Four instruments were used: a demographic data sheet, the Perceived Organizational Support Scale, the Role Clarity and Role Ambiguity Questionnaire, and the Career Plateauing Scale. All instruments demonstrated acceptable reliability and validity in previous nursing studies.

(1) Personal characteristics data sheet: A self-administered questionnaire created by the researchers and including nurse age, sex, marital status, educational qualification, years of experience, and the hospital where they worked.

(2) Organizational Support Scale: Developed by Eisenberger et al. [12], this assesses perceived support from the organization. This tool includes 17 items (8 items that measure an employee’s perceptions of the degree to which the organization values the worker’s contributions and 9 items about actions that the organization might take that would affect the well-being of the employee) rated on a 7-point Likert scale, ranging from strongly disagree (1) to strongly agree (7). Cronbach’s alpha = 0.89. Score interpretation varies according to the 17 items, with the total score equaling 119. Total scores range from 17 to 119, with higher scores indicating higher perceived organizational support. Scores from 17 to 51 indicate low support, 52–85 moderate support, and 86–119 high perceived organizational support.

(3) Role Clarity and Ambiguity Questionnaire: Developed by [13], it comprises 28 items, 6 measuring nurse’s perceptions about role clarity via a scoring system (disagree = 1, uncertain = 2, and agree = 3). A further 22 items measure the causes of role ambiguity (yes = 2 and no = 1). Score interpretation varies according to the 6 items, with a total score equaling 18. Scores from 6 to 10 indicate low role clarity, scores from 11 to 14 indicate moderate role clarity, and scores from 15 to 18 indicate high role clarity. Cronbach’s alpha for clarity = 0.84; ambiguity = 0.87.

Although the original description by [13] referred to the 22 items as measuring the causes of role ambiguity, each statement actually represents a situation that reflects a nurse’s degree of perceived ambiguity in performing professional duties. Therefore, consistent with previous nursing studies that applied this instrument in Egypt, e.g., [14,15], the total score of these 22 items was interpreted as an indicator of the extent to which nurses experience role ambiguity in their daily work. Higher total scores correspond to greater perceived ambiguity, and the total score was therefore included in the correlation and regression analyses as a quantitative measure of role ambiguity.

(4) Career Plateauing Scale: It was developed by [16] to indicate the extent to which staff nurses experience feelings and perception regarding career plateau. It contains 20 items across two domains: structural/hierarchical has “9 items” and job content has “11 items”. Each item is answered on a 7-point Likert scale ranging from 1 point (strongly disagree) to 7 points (strongly agree). Score interpretation varies according to the 20 items, with the total score equaling 140. Scores from 20 to 63 indicate low career plateauing, scores from 64 to 100 indicate moderate career plateauing, and scores from 101 to 140 indicate high career plateauing.

Validity and Reliability of study tools

After reviewing the relevant literature on the research topic, the study tools were translated into Arabic. The face validity of these tools was assessed by five experts in the field at the same academic institution.

To test reliability, Cronbach’s alpha coefficient was employed, resulting in coefficients of α = 0.821 for the Organizational Support Scale, α = 0.858 for the Role Clarity Scale, α = 0.799 for the Role Ambiguity Scale, and α = 0.798 for the Career Plateauing Scale. These values indicate a high level of reliability of the study tools.

The psychometric properties of the study instruments were supported by previous studies that demonstrated their acceptable reliability and construct validity in nursing and organizational research. The Perceived Organizational Support Scale developed by Eisenberger et al. [12] has been widely validated across diverse healthcare contexts, showing Cronbach’s alpha values ranging from 0.84 to 0.93 in prior studies. Similarly, the Role Clarity and Role Ambiguity Questionnaire was previously applied among Egyptian nursing samples, confirming its cultural relevance and internal consistency. The Career Plateauing Scale developed by Bardwick [16] has also been supported by subsequent research reporting reliability coefficients above 0.80 (e.g., [17,18]). These references collectively confirm the strong psychometric foundation of the instruments used in the current study.

Pilot study

A pilot study was carried out with 10% of the participants (21 nurses) to test the clarity, feasibility and applicability of the study tools, to determine the time required to fill out the questionnaires and to explore any obstacles that the researchers may encounter during data collection.

Field work

The researchers distributed the study questionnaires directly to the participants. Each nurse received a hand-delivered copy from the researchers, who later collected the completed forms. The purpose of the study was explained to each nurse individually in a brief, two-minute discussion before they were asked to complete and return the questionnaire. To ensure objectivity, thoughtful responses, and full completion, the nurses filled out the forms in the presence of the researcher. Since the participants were assigned to specific work units, it was easy to track questionnaire distribution and collection, helping to achieve a high response rate. As a token of appreciation, participants were given small snacks. Completing the questionnaire took approximately 20 to 25 min. Data collection occurred over a two-month period from September to October 2025. Researchers also addressed all participant inquiries and provided necessary clarifications.

Ethical Considerations

Ethical approval was obtained from the Faculty of Nursing Ethics Committee (approval no. 1120251191). Participation was voluntary, informed consent was obtained, and confidentiality was assured.

Statistical Analysis

Data were analyzed using IBM SPSS Statistics version 27. Descriptive statistics were used to summarize participants’ characteristics, with categorical variables presented as frequencies and percentages (N, %) and continuous variables described using means and standard deviations (Mean ± SD).

Prior to inferential analyses, data were assessed for normality using the Anderson–Darling test and for homogeneity of variances. Comparisons of continuous variables across demographic groups were conducted using independent samples *t*-tests or one-way analysis of variance (ANOVA), as appropriate.

Pearson correlation coefficients were calculated to examine the relationships among organizational support, role clarity, role ambiguity, and career plateauing scores. Univariate and multivariate linear regression analyses were performed to identify predictors of nurses’ perceptions of career plateauing. All statistical tests were two-tailed, and a *p*-value < 0.05 was considered statistically significant.

## 3. Results

A total of 210 nurses completed the questionnaire and were included in the final analysis. The demographic profile in Table 1, shows that more than half of the participants were under 30 years (51% under 30 years), female (81%), and predominantly married (71.9%). The majority held a bachelor’s degree or technical nursing qualification. Notably, 64.3% had less than 10 years of experience, indicating a relatively youthful and moderately experienced nursing workforce. This demographic pattern may influence nurses’ perceptions of career development, particularly with regard to their expectations for advancement and organizational support.

The results indicate that nurses reported moderate organizational support (Mean = 64.54, 54.23%), high role ambiguity (Mean = 32.23, 73.25%), and high role clarity (Mean = 14.55, 80.83%) (Table 2). Role ambiguity was particularly notable, reflecting that many nurses experienced unclear responsibilities and expectations. Both hierarchical plateauing (Mean = 36.27, 57.57%) and job content plateauing (Mean = 48.60, 63.12%) were moderate, resulting in an overall career plateauing score of 84.87 (60.62%). These findings suggest that, despite a generally good understanding of their roles, nurses experience substantial ambiguity alongside moderate organizational support, which may contribute to perceptions of career plateauing in both hierarchical and job content domains.

**Figure 1:**
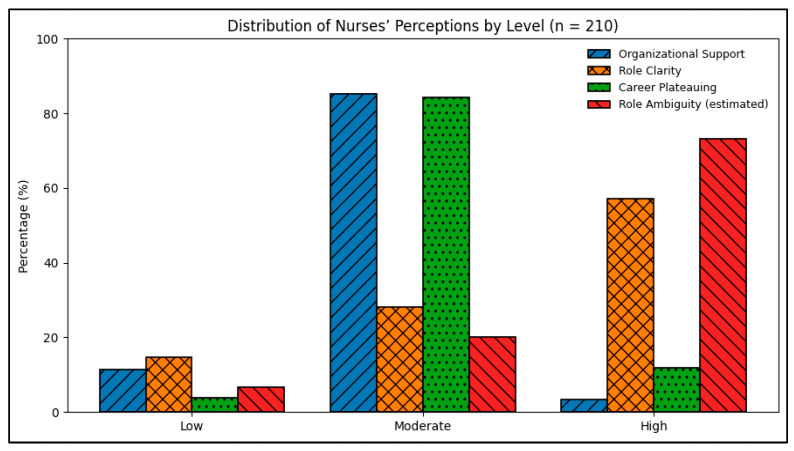
Distribution of nurses’ perceptions of organizational support, role clarity, role ambiguity, and career plateauing by level (n = 210).

Figure 1 presents the distribution of nurses’ perceptions of organizational support, role clarity, and career plateauing by level. The majority of nurses perceived a moderate level of organizational support (85.2%), while smaller proportions reported low (11.4%) or high (3.3%) support, indicating that although basic support is available, it is rarely perceived as optimal. Regarding role clarity, more than half of the nurses (57.1%) reported high clarity, followed by 28.1% reporting moderate clarity and 14.8% low clarity, suggesting that most nurses understand their job responsibilities, although a notable minority continue to experience role-related challenges. With respect to career plateauing, most nurses (84.3%) reported moderate levels, while 11.9% experienced high plateauing and only 3.8% reported low levels, reflecting a widespread perception of limited career progression that may adversely affect motivation, job satisfaction, and retention.

In contrast, role ambiguity was predominantly high, indicating that many nurses’ experience uncertainty related to role boundaries, responsibilities, or expectations despite having general role clarity. This coexistence of high role clarity and high role ambiguity reflects the complexity of nursing roles in dynamic hospital environments, where formal job descriptions may be clear but daily practice involves overlapping tasks and evolving demands. Overall, the figure highlights the imbalance between structured organizational roles and perceived career growth opportunities among staff nurses.

The correlation analysis (Table 3) reveals a statistically significant negative relationship between role ambiguity and both role clarity (r = −0.337) and career plateauing (r = −0.441), suggesting that increased role ambiguity is associated with lower perceptions of career plateauing. This indicates that ambiguous roles may introduce variability or perceived flexibility that reduces feelings of plateauing.

Conversely, role clarity and organizational support show a positive correlation with career plateauing, indicating that nurses who perceive greater support and clarity also report less plateauing. These findings emphasize the detrimental impact of ambiguity and the buffering role of clarity and support on career development perceptions.

The multivariate regression analysis in Table 4 identifies role ambiguity as a statistically significant negative predictor of hierarchical plateauing (β = −0.285, *p* < 0.001), suggesting that increased ambiguity strongly contributes to feelings of plateauing in hierarchical growth. In contrast, organizational support (β = 0.016, *p* = 0.812) and role clarity (β = 0.122, *p* = 0.082) were not statistically significant predictors in the model. The overall model explained approximately 12.2% of the variance in hierarchical plateauing (R^2^ = 0.122), with a significant F-value of 9.52, indicating a meaningful model fit.

This regression model (Table 5) shows that all three predictors—organizational support, role ambiguity, and role clarity—significantly influence job content plateauing. Specifically, organizational support (β = 0.193, *p* = 0.003) and role clarity (β = 0.190, *p* = 0.005) positively predict job content plateauing, suggesting that when support and clarity are higher, nurses report greater awareness of stagnant job roles. Role ambiguity (β = −0.267, *p* < 0.001) negatively predicts job content plateauing, indicating that higher ambiguity reduces perceptions of plateauing. The model accounts for approximately 20.9% of the variance (R^2^ = 0.209), with an F-value of 18.18, indicating a strong model fit.

In Table 6, the final regression model demonstrates that all three independent variables significantly contribute to predicting nurses’ perceptions of overall career plateauing. Role ambiguity remains the strongest negative predictor (β = −0.350, *p* < 0.001), reinforcing its central role in plateauing perceptions. Meanwhile, both organizational support (β = 0.140, *p* = 0.024) and role clarity (β = 0.200, *p* = 0.002) positively predict plateauing awareness, possibly indicating heightened expectations among nurses who perceive support and clear roles. The model explains 25.3% of the variance in career plateauing (R^2^ = 0.253), and the significant F-value (23.31) indicates good model fit.

This analysis shows significant differences in perceived organizational support based on age, marital status, educational qualification, and years of experience. Older nurses and those with more experience reported significantly higher support, possibly due to increased seniority or familiarity with organizational structures. Additionally, married nurses and those with higher education levels also perceived greater support, which could reflect greater integration or status within the institution. No significant differences were observed based on gender, as shown in Table 7. ANOVA followed by Tukey’s HSD post hoc tests was applied to determine between-group differences. Significant differences were observed for age, marital status, educational qualification, and years of experience.

## 4. Discussion

The current findings should be interpreted within a broader context encompassing nursing organization and workforce dynamics, particularly in developing healthcare systems where career structures may be less flexible than in Western systems. In contrast to studies conducted in Europe and North America, where career progression and specialization pathways are more established, Egyptian university hospitals often operate within centralized promotion systems that may slow advancement opportunities. Consequently, perceptions of career plateauing may emerge early in a nurse’s career despite moderate organizational support. Similar patterns have been reported in international studies linking limited promotion structures to increased perceptions of career plateauing among healthcare workers.

Studies conducted within the National Health Service in the UK, Australian public hospitals, Canadian regional health authorities, and several EU healthcare systems have similarly identified rigid hierarchical promotion structures, limited leadership pathways, and insufficient professional development programs as structural contributors to career plateauing among nurses. However, unlike many Western systems where structured specialty tracks and lateral mobility options exist, Egyptian university hospitals operate within more centralized and seniority-based advancement frameworks. This difference may explain why, in the present study, organizational support did not significantly reduce hierarchical plateauing and, in some cases, was positively associated with plateau perceptions. These findings suggest that in highly centralized systems, formal support mechanisms may enhance role clarity without necessarily expanding real promotion opportunities [18,19].

This study highlights the significant impact of role-related factors and organizational support in shaping nurses’ perceptions of career plateauing. Role ambiguity emerged as the strongest predictor of both hierarchical and job content plateauing, underscoring the importance of clear role expectations in mitigating feelings of career plateauing. These findings align with recent international nursing studies indicating that unclear roles contribute to stress, disengagement, and limited professional growth among hospital nurses.

Career plateauing and the absence of effective career development programs have been shown to substantially reduce employee motivation and performance, with adverse consequences for both individuals and the quality of public services delivered. Accordingly, managerial interventions are essential to address these challenges through supportive organizational policies, such as expanded access to training, performance-based incentives, and transparent promotion systems, which collectively enhance employee morale and professional engagement [20].

In addition to structural reforms, recent evidence suggests that individualized professional interventions can help alleviate career plateauing. Ref. [21] demonstrated that a practical, methodology-based life design counseling program significantly improved career adaptability and reduced plateauing among mid-career professionals. While such programs are typically implemented outside of healthcare settings, they may be particularly beneficial in teaching hospitals, where formal advancement pathways are often limited. Therefore, integrating career guidance frameworks into nursing management practices would complement organizational reforms by fostering a sense of agency and career planning among nurses.

Recent integrative evidence synthesizing domestic and international empirical studies has further demonstrated that career plateauing is consistently associated with lower organizational commitment, reduced engagement, and higher turnover intentions [18]. These findings reinforce the practical significance of the present results, as moderate levels of plateauing among nurses in university hospitals may have long-term implications for workforce stability and retention.

Recent empirical evidence further underscores the psychological consequences of career plateauing. A study by [22] demonstrated that career plateauing significantly predicts job burnout, with diminished career calling mediating this relationship. In high-demand healthcare settings such as university hospitals, where emotional labor and workload pressures are substantial, sustained perceptions of plateauing may therefore exacerbate exhaustion and disengagement. The moderate levels of plateauing identified in the present study are thus not merely structural concerns but may also signal potential risks for nurse well-being and service quality.

The present study examined nurses’ perceptions of organizational support, role clarity, and role ambiguity and their influence on career plateauing. The demographic profile revealed that most participants were female, married, under 30 years of age, had between 1 and 10 years of professional experience, and held a Bachelor of Nursing Science degree. This profile reflects the gendered nature of the nursing profession and aligns with sociocultural norms related to early career entry, family formation, and workforce composition within nursing roles.

The predominance of younger, female nurses in the present sample may shape perceptions of career plateauing. However, this pattern likely reflects cohort and career-stage effects rather than universal career trajectories. Early-career nurses often expect rapid advancement and diverse professional development opportunities [23,24,25]; when clear promotion pathways, mentorship, and structured career ladders are limited, perceptions of plateauing may intensify. Previous research has emphasized that career plateauing is strongly influenced by career stage, promotion expectations, and perceived mobility opportunities within organizations [19]. In contrast, many Western healthcare systems have older and more tenure-based nursing workforces, where plateauing may emerge later in the professional trajectory.

These findings highlight the importance of age- and experience-sensitive retention strategies, including structured early-career development programs, transparent promotion criteria, mentorship initiatives, and flexible career pathways. Such approaches may help mitigate dissatisfaction, reduce turnover intentions, and support sustainable workforce development across nursing cohorts.

The majority of nurses reported moderate levels of perceived organizational support, indicating that while foundational support mechanisms exist, they remain insufficient to fully meet nurses’ professional expectations. Despite the availability of staffing, training, and communication resources, factors such as heavy workloads, limited recognition, and inconsistent managerial engagement may undermine nurses’ perceptions of support. These findings are consistent with previous studies reporting moderate organizational support among nursing staff [26,27].

More than half of the nurses experienced moderate role clarity, suggesting an incomplete understanding of role boundaries and expectations. This may be attributed to overlapping duties, evolving clinical protocols, and inadequate communication. Outdated job descriptions and task expansion beyond formal responsibilities, particularly in understaffed or high-pressure environments, may further exacerbate role-related challenges. These findings are consistent with earlier research reporting similar levels of role clarity [14], although they contrast with studies identifying higher clarity levels among nurses [15].

Career plateauing was reported at moderate levels by most nurses, reflecting prolonged periods without promotion, repetitive job responsibilities, and limited opportunities for professional growth within rigid organizational hierarchies. Such conditions are known to diminish motivation and job satisfaction and reinforce perceptions of career plateauing. These findings align with previous studies that reported similar patterns of plateauing linked to limited recognition and advancement opportunities [8,28].

A comprehensive review of four decades of research on career path plateauing, conducted by [19], confirms that this plateauing is shaped not only by individual characteristics but also by organizational structural constraints, role design, and available career mobility opportunities. The review distinguishes between hierarchical plateauing, reflecting limited promotion opportunities, and job content plateauing, associated with monotonous tasks and underutilization of skills. The current findings support this multidimensional view, particularly in demonstrating the contrasting effects of role ambiguity and organizational support on both forms of plateauing.

The high mean score of the career plateauing scale further underscores nurses’ perceptions of professional plateauing associated with restricted promotion pathways, role monotony, and insufficient support for continuing education. Comparable findings have been reported in prior studies examining barriers to career development among nursing staff [29,30].

Correlation analysis demonstrated that perceived organizational support was positively associated with both career plateauing and role clarity. While organizational support may enhance nurses’ understanding of their roles and improve job satisfaction, it may also increase awareness of limited advancement opportunities, thereby intensifying perceptions of plateauing. This dual effect of organizational support has been highlighted in previous research, particularly in settings where career progression opportunities are constrained [31].

Multivariate analysis identified role ambiguity as the strongest predictor of hierarchical career plateauing, with higher ambiguity associated with greater perceptions of plateauing in upward mobility. Contrary to traditional expectations, role ambiguity demonstrated a negative relationship with career plateauing. One possible explanation is that ambiguous roles may allow for greater task variation and informal learning opportunities, thereby reducing perceptions of career plateauing despite organizational uncertainty.

In contrast, organizational support showed no significant effect, and role clarity exerted only a marginal positive influence. These findings suggest that unclear responsibilities and expectations hinder nurses’ perceptions of promotion opportunities, while organizational support alone is insufficient to facilitate career advancement. These results are consistent with prior studies emphasizing the central role of role ambiguity in shaping career outcomes [32,33].

Regarding job content plateauing, role ambiguity negatively predicted plateauing, indicating that greater ambiguity may allow for flexibility, task variation, and perceived learning opportunities, thereby reducing feelings of plateauing. Conversely, organizational support and role clarity positively predicted job content plateauing, possibly reflecting the constraining effects of highly structured roles that limit opportunities for job enrichment. These findings support conclusions reported in earlier studies [34,35].

Similarly, organizational support and role clarity were positively associated with overall career plateauing perceptions, whereas role ambiguity exerted a negative effect. Structured and supportive work environments may stabilize nursing roles but inadvertently restrict opportunities for growth, whereas some degree of ambiguity may facilitate broader role engagement and perceived professional development. These findings correspond with previous research [8,36].

Finally, demographic variables such as age, marital status, and years of experience significantly influenced perceptions of organizational support. Older nurses, married individuals, and those with greater professional experience reported higher perceived support, likely reflecting stronger professional integration, recognition, and access to work–life balance accommodations. These associations are supported by earlier studies [9,37,38].

### Study Limitations

This study offers important insights into the relationship between organizational support, role clarity and ambiguity, and career stagnation among nurses. However, several limitations should be considered. First, the descriptive correlational design limits the possibility of establishing a causal relationship between variables. Although statistically significant correlations were identified, the study cannot prove causal relationships.

Second, the sample was limited to two university hospitals in Egypt, which may limit the generalizability of the findings to other healthcare institutions, such as private hospitals or institutions with different administrative or organizational structures. Third, the study relied on self-administered questionnaires, which may be susceptible to response biases, including social desirability bias and inaccuracy of self-assessment, potentially influencing participants’ perceptions of organizational support and role-related factors.

Furthermore, the nurses were not asked to explain the reasons behind their responses. Therefore, these findings represent a preliminary exploration of nurses’ perceptions of career stagnation and related organizational factors, rather than an in-depth analysis of the factors influencing these perceptions. Future studies should employ qualitative or mixed approaches to better understand the reasons behind nurses’ responses and provide deeper insight into the mechanisms contributing to career stagnation.

Finally, the cross-sectional nature of this study captures perceptions at a specific point in time and does not reflect changes over time or the dynamic nature of career development. Furthermore, despite the use of validated instruments, the cultural and contextual factors specific to the Egyptian healthcare environment may influence the interpretation of concepts such as role ambiguity and organizational support, potentially limiting the applicability of the findings to other cultural or healthcare contexts. Future research using longitudinal designs, larger and more diverse samples, and mixed approaches is recommended to further validate and expand the scope of these findings across different healthcare settings.

## 5. Conclusions

This study highlights the significant impact of organizational support, role clarity, and role ambiguity on nurses’ experiences of career plateauing at Al Rajhi Hospitals and University Heart Hospital. Among the variables studied, role ambiguity emerged as the strongest predictor of career plateauing, followed by role clarity and organizational support. A significant positive relationship was observed between organizational support, role clarity, and career plateauing, while role ambiguity showed a significant negative relationship with organizational support, role clarity, and career plateauing. These findings underscore the need for healthcare organizations to address role uncertainty and strengthen support structures to prevent career plateauing among nurses.

## 6. Recommendations

Based on the study findings, organizational-level interventions are recommended to reduce career plateauing and improve professional development opportunities within university hospitals. Also, they should provide comprehensive orientation and ongoing training for nurses to ensure role clarity and reduce career ambiguity. Additionally, they should provide career guidance and counseling services to help nurses explore personal development goals and long-term career opportunities.

Moreover, we recommend improving communication and leadership practices by setting specific, measurable goals and providing clear guidelines on performance standards and work procedures. Furthermore, it is important to standardize and clarify job descriptions and performance expectations early upon hiring to reduce role ambiguity and support professional growth from the outset.

## Figures and Tables

**Table 1:** Distribution of demographic data of nurses working at Al-Rajhi & Heart University Hospitals (n = 210).

Variable	Category	No.	%
Hospital	Heart Hospital	110	52.3
	Al-Rajhi Hospital	100	47.6
Age Group	Less than 30 years	107	51.0
	30–40 years	82	39.0
	More than 40 years	21	10.0
	Mean ± SD (Range)		32.17 ± 5.62 (19–52)
Sex	Male	40	19.0
	Female	170	81.0
Marital Status	Single	57	27.1
	Married	151	71.9
	Divorced	1	0.5
	Widow	1	0.5
Educational Qualification	Nursing Secondary School	53	25.2
	Bachelor of Nursing Science	73	34.8
	Specialist Nursing School	18	8.6
	Nursing Institute	66	31.4
Years of Experience	5 to <10 years	135	64.3
	10 to <15 years	55	26.2
	>20 years	20	9.5
	Mean ± SD (Range)		10.47 ± 6.06 (5–30)

**Table 2:** Mean scores of Organizational Support, Role Ambiguity, Role Clarity, and Career Plateauing Scales (n = 210).

Scale	Max Score	Mean ± SD	Range	Mean%
Organizational Support	119	64.54 ± 12.06	31–98	54.23%
Role Ambiguity	44	32.23 ± 4.53	22–44	73.25%
Role Clarity	18	14.55 ± 3.21	7–18	80.83%
Hierarchical Plateauing	63	36.27 ± 7.91	12–60	57.57%
Job Content Plateauing	77	48.60 ± 9.24	13–74	63.12%
Career Plateauing (Total)	140	84.87 ± 13.61	25–122	60.62%

**Table 3:** Correlation coefficients between organizational support, role ambiguity, role clarity, and career plateauing (n = 210).

Variable	Org. Support	Role Ambiguity	Role Clarity
Role Ambiguity	−0.189 **	1	
Role Clarity	0.154 *	−0.337 **	1
Career Plateauing	0.236 **	−0.441 **	0.338 **

Note: * *p* < 0.05; ** *p* < 0.01.

**Table 4:** Multivariate linear regression model predicting hierarchical plateauing from organizational support, role ambiguity, and role clarity (n = 210).

Predictor Variable	Beta	t	Sig.	95% CI	R^2^	F
Organizational Support	0.016	0.239	0.812	−0.08 to 0.10		
Role Ambiguity	−0.285	−4.062	0.000 **	−0.74 to −0.26		
Role Clarity	0.122	1.748	0.082	−0.04 to 0.64	0.122	9.52

Dependent variable: hierarchical plateauing; statistically significant at ** *p* < 0.01.

**Table 5:** Multivariate linear regression model predicting job content plateauing from organizational support, role ambiguity, and role clarity (n = 210).

Predictor Variable	Beta	t	Sig.	95% CI	R^2^	F
Organizational Support	0.193	3.046	0.003 **	0.05 to 0.24		
Role Ambiguity	−0.267	−4.012	0.000 **	−0.81 to −0.28		
Role Clarity	0.190	2.871	0.005 **	0.17 to 0.92	0.209	18.18

Dependent variable: job content plateauing; statistically significant at ** *p* < 0.01.

**Table 6:** Multivariate linear regression model predicting career plateauing from organizational support, role ambiguity, and role clarity (n = 210).

Predictor Variable	Beta	t	Sig.	95% CI	R^2^	F
Organizational Support	0.140	2.280	0.024 *	0.02 to 0.30		
Role Ambiguity	−0.350	−5.360	0.000 **	−1.42 to −0.66		
Role Clarity	0.200	3.110	0.002 **	0.31 to 1.38	0.253	23.31

Dependent variable: Career Plateauing Scale; statistically significant at * *p* < 0.05; statistically significant at ** *p* < 0.01.

**Table 7:** Comparison of organizational support scores across nurses’ demographic variables (n = 210).

Demographic Variable	N	Mean ± SD	Range	Test Used	*p*-Value
Age Group				One-way ANOVA → Tukey HSD: >40 yrs. > <30 & 30–40 yrs.	0.010 *
Less than 30 years	107	63.5 ± 12.58	31–98		
30–40 years	82	63.96 ± 11.22	31–85		
More than 40 years	21	72.05 ± 10.25	43–87		
Sex				*t*-test	0.822
Male	40	64.93 ± 11.10	47–92		
Female	170	64.45 ± 12.30	31–98		
Marital Status				One-way ANOVA → Tukey HSD: Married > Single	0.005 **
Single	57	60.54 ± 11.08	31–87		
Married	151	66.17 ± 12.04	31–98		
Divorced/Widow	2	55.00 ± 16.97	43–67		
Educational Qualification				One-way ANOVA → Tukey HSD: Secondary School > Institute	0.024 *
Nursing Secondary School	53	67.47 ± 12.33	31–98		
Bachelor Degree	73	65.84 ± 10.78	40–85		
Specialist Nursing School	18	62.72 ± 14.86	32–88		
Nursing Institute	66	61.24 ± 11.77	31–92		
Years of Experience				One-way ANOVA → Tukey HSD: >20 yrs. > 5–10 & 10–20 yrs.	0.005 **
5–<10 years	135	63.53 ± 12.31	31–98		
10–<15 years	55	64.00 ± 11.18	42–87		
>20 years	20	72.80 ± 9.81	43–84		

Statistically significant at * *p* < 0.05; statistically significant at ** *p* < 0.01.

## Data Availability

Data supporting the findings of this study are available from the corresponding author upon reasonable request. The data are not publicly available due to in accordance with ethical approval and participant confidentiality.
